# The Value of Anlotinib in the Treatment of Intractable Brain Edema: Two Case Reports

**DOI:** 10.3389/fonc.2021.617803

**Published:** 2021-03-22

**Authors:** Song Yang, Jian Sun, Mingna Xu, Yuru Wang, Guihong Liu, Aijun Jiang

**Affiliations:** Center of Clinical Oncology, Affiliated Hospital of Xuzhou Medical University, Xuzhou, China

**Keywords:** anlotinib, peritumoral brain edema (PTBE), intractable, radiotherapy, vascular endothelial growth factor receptor (VEGFR)

## Abstract

About 20-30 percent of patients with cancer, such as non-small cell lung cancer, breast cancer, melanoma and renal cell carcinoma, will develop brain metastases (BM). Primary and secondary brain tumors are often accompanied by peritumoral edema. Due to the limited intracranial space, peritumoral edema will further increase the intracranial pressure and aggravate clinical symptoms. Radiotherapy, as a basic component of the treatment of intracranial tumors, induces blood vessel damage and aggravates brain edema. The combination of edema caused by the tumor itself and radiotherapy is collectively referred to as intractable brain edema. Edema can increase intracranial pressure and cause associated neurologic symptoms, which seriously affects the quality of life of patients. Steroids, specifically dexamethasone, have become the gold standard for the management of tumor-associated edema. However, steroids can lead to variety of adverse effects, including moon face, high blood pressure, high blood sugar, increased risk of infection, bone thinning (osteoporosis), and fractures, especially with prolonged use. The investigation of other types of drugs is urgently needed to address this problem.Compared to other anti-angiogenic agents, anlotinib acts on vascular endothelial growth factor receptors (VEGFR1, VEGFR2/KDR, and VEGFR3), fibroblast growth factor receptors (FGFR1, FGFR2, FGFR3 and FGFR4), platelet derived growth factor receptor (PDGFR) and stem cell factor receptor (c-kit) simultaneously. However, according to the literature retrieval, there are no studies on anlotinib for the treatment of intractable brain edema. We describe here two cases of brain edema and review the literature available and hope to discover new agents that are safer and more effective.

## Introduction

Up to 20–40% of patients with cancer, such as non-small cell lung cancer, breast cancer, melanoma, and renal cell carcinoma, will develop brain metastases (BM) (1–3). Primary and secondary brain tumors are often accompanied by peritumoral edema. Due to the limited intracranial space, peritumoral edema will further increase the intracranial pressure and aggravate clinical symptoms. Radiotherapy, as a basic component of the treatment of intracranial tumors, induces blood vessel damage and aggravates brain edema (4). The blood brain barrier can be damaged by tumor and radiotherapy which allows increased passage of plasma proteins and water into the extracellular compartment (5, 6). In addition, angiogenic edema resulting from an increase of vascular permeability plays a key role in the development of intractable brain edema (7). The combination of edema caused by the tumor itself and radiotherapy is collectively referred to as intractable brain edema. Edema can increase intracranial pressure and cause associated neurologic symptoms, which seriously affects the quality of life of patients. Claudin and occludin can be downregulated by glucocorticoids in vasogenic edema to decrease capillary permeability and extravasation of fluid (8). Meanwhile, it can also decrease extent of cytokine-driven blood-brain barrier breakdown *via* suppressing pro-inflammatory transcription factor NF-B and proinflammatory cytokines (9). Therefore, glucocorticoids, specifically dexamethasone, have become the gold standard for the management of tumor-associated edema (10). However, glucocorticoids can lead to variety of adverse effects, including moon face, high blood pressure, high blood sugar, increased risk of infection, bone thinning (osteoporosis), and fractures, especially with prolonged use (11). The investigation of other types of drugs is urgently needed to address this problem.

Compared to other anti-angiogenic agents, anlotinib acts on vascular endothelial growth factor receptors (VEGFR1, VEGFR2/KDR, and VEGFR3), fibroblast growth factor receptors (FGFR1, FGFR2, FGFR3, and FGFR4), platelet derived growth factor receptor (PDGFR) and stem cell factor receptor (c-kit) simultaneously (12) (**Table 1**). As a multi-target small molecule antiangiogenic agent, it can cross the blood-brain barrier and inhibit multiple signaling pathways including PI3K/AKT, MAPK/ERK, and RAF/MRK to inhibit both tumor angiogenesis and tumor cell proliferation (12–16). However, according to the literature retrieval, there are no studies on anlotinib for the treatment of intractable brain edema. We describe here two cases of brain edema and review the literature available and hope to discover new agents that are safer and more effective.

**Table 1:** The different targets between anlotinib and other agents.

	VEGFR			PDGFR		FGFR				c-kit
	1	2	3	α	β	1	2	3	4	
Anlotinib	**+**	**+**	**+**	**+**	**+**	**+**	**+**	**+**	**+**	**+**
Nintedanib	**+**	**+**	**+**	**+**	**+**	**+**	**+**	**+**	**-**	**-**
Pazopanib	**+**	**+**	**+**	**+**	**+**	**+**	**-**	**-**	**-**	**+**
Sunitinib	**+**	**+**	**+**	**+**	**+**	**-**	**-**	**-**	**-**	**+**
Apatinib	**+**	**+**	**+**	**-**	**+**	**+**	**-**	**-**	**-**	**+**
Vatalanib	**+**	**+**	**+**	**-**	**+**	**-**	**-**	**-**	**-**	**+**
Sorafenib	**+**	**+**	**+**	**-**	**+**	**-**	**-**	**-**	**-**	**+**
Axitinib	**+**	**+**	**+**	**-**	**-**	**-**	**-**	**-**	**-**	**-**
Bevacizumab	**+**	**+**	**-**	**-**	**-**	**-**	**-**	**-**	**-**	**-**

+, target; -, no target.

## Case 1

A 66-year-old Chinese male presented to a hospital in February 2020 with several months of episodic coughing and night sweats. A lesion (56×55 mm) in the right upper lung lobe was detected by computed tomographic (CT) imaging. The needle biopsy demonstrated non-small cell lung cancer with immunohistochemical results of Ki67(+, 70%), TTF(-), CD56(-), Syn(-), CgA(-), LCA(-), P40(+/-), CK5/6(+), CK(+), Napsin(-), CKpan(+), and P63(+/-), with a tendency to adenocarcinoma. Molecular analysis showed only *KRAS* gene mutations and no mutations in the *EGFR, ROS1, RET, HER2, BRAF, NRAS*, or *ALK*. PD1 and PDL1 expression levels were not investigated due to patient’s economic situation. Systemic evaluation showed the involvement of multiple lymph nodes (right supraclavicular region, mediastinum, and bilateral hilum of the lung), and possible brain metastases (5×7 mm) in the left frontal lobe. At this stage, the patient underwent chemotherapy (two cycles of TP: Paclitaxel and Nedaplatin).

**Figure 1:**
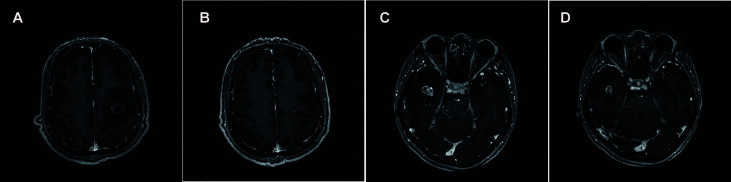
Brain MRIs of the patient revealed the size of BM lesion and PTBE. Comparing with the brain MRI before, the maximum vertical diameter of BM demonstrated slight shrunk (17×22 to 14×18 mm) **(A, B)** and (15×17 to 12×9 mm) **(C, D)** while peritumoral brain edema shrunk significantly.

In May 2020, he complained of headaches and fatigue. Investigation showed that the lesion has enlarged to 17×22 mm and peritumoral edema in the left frontal lobe was found by postaxial T1 weighted magnetic resonance imaging (MRI). Local radiotherapy with a dose of 2Gy/f*15f to the single BM was administered after nine days of dehydration treatment (mannitol: 250 ml, 6 am, 4 pm; glycerol fructose: 250 ml, 12 n, 12 mn; hexadecadrol: 3 mg, qd). However, the patient complained of recurring headaches and fatigue that showed no improvement after 12 days of conventional intervention with dehydration and 4 days of radiotherapy. He was taken treated with anlotinib (12 mg per day, days 1–14; 21 days per cycle) and continued with steroids administration. After 10 days of anlotinib treatment, his condition was assessed again. Surprisingly, the symptoms of headache and fatigue were significantly relieved. Meanwhile, compared with the previous brain MRI, the maximum vertical diameter of the BM showed slight shrunk (from 17×22 to 14×18 mm) while the peritumoral brain edema had reduced significantly (**Figures 1A, B**).

## Case 2

A 32-year-old female with headache of two months’ duration was admitted to our hospital in July 2017. MRI of the brain revealed multiple abnormal signals, with the largest lesion (27×41×27 mm) was located in the left cerebellar hemisphere. Chest CT revealed a mass (41×40 mm) in the lower lobar region of the left lung. Lung cancer with multiple brain metastases was suspected. Subsequently, the largest one was surgically removed to release the compressed fourth ventricle. Pathology revealed a poorly differentiated metastatic adenocarcinoma, and the *EGFR* mutation 19-Del was detected. After six cycles of chemotherapy-with gefitinib (Pemetrexeddisodium, PEM 1.0 g+ Nedaplatin, NDP 130 mg; gefitinib 250 mg qd) by sequential therapy, the response was evaluated as stable disease (SD). In August 2018, CT revealed enlargement of the left lower lobar lesion. One cycle of chemotherapy (PEM 1.0 g) was again undertaken. One month later, however, bone metastases appeared. After three cycles of chemotherapy (PEM 1.0 g+ NDP 120 mg), the lesion in the inferior lobe of the left lung had slightly increased compared to before. She refused the next cycle of chemotherapy and started taking Osimertinib (80 mg qd) instead of gefitinib.

In January 2020, MRI of the brain revealed a single new metastatic lesion (5×5 mm) on the right side of the temporal lobe. Two months later, the new metastatic lesion (11×10×13 mm) was found to have enlarged. Subsequently, she underwent one cycle of chemotherapy (PEM 1.0 g+ CBP 400 mg). At the end of April, she complained of headache. The enlarged lesion (15×17 mm) and peritumoral brain edema on the right side of the temporal lobe were observed on MRI. At this stage, the patient received the conventional intervention of dehydration (mannitol: 250 ml bid; glucocorticoid: 3 mg qd) and local radiotherapy (Phase I: 2.0Gy/f*19f; Phase II: 3.0Gy/f*4f) was administered to the single BM. However, she experienced no noticeable relief from headaches after a week. Oral administration of anlotinib was initiated (12 mg per day, days 1–14; 21 days per cycle) and continued with steroids administration. Two weeks later, surprisingly, compared with the previous brain MRI, the BM demonstrated showed slight shrinkage (15×17 mm to 12×9 mm) while the peritumoral edema has shrunk significantly (**Figures 1C, D**).

## Discussion

Angiogenic edema plays a key role in the occurrence and development of intractable brain edema. Vascular endothelial growth factor (VEGF) can significantly increase the vascular permeability of the tumor (17). Nassehi et al. (18) found that the peritumoral edema index was positively correlated with the expression of the VEGF gene and VEGF-A protein. Blocking the VEGF pathway can decrease vascular permeability and, thus, cerebral edema (7). Although glucocorticoids are traditionally used for the treatment of intractable brain edema, they have multiple side effects, relatively poor safety and efficacy, and are unable to inhibit the progression of brain tumors (19). In addition, with the introduction of immune-checkpoint inhibitors (including PD-1/PD-L1 and CTLA-4) in clinical practice, the research of alternatives to steroids remains a critical issue. The etiology of cerebral oedema in patients treated with immunotherapy is unclear, because it may be difficult to distinguish disease progression from pseudoprogression (20). For the treatment of immune-related neurological adverse events, the use of systemic steroids may be recommended, accompanied by either a delay or cessation of checkpoint inhibitor therapy. There are few alternatives to steroids for this indication.

Anti-angiogenic agents, which have been increasingly used in cancer patients with progressive malignant brain tumors, can improve the tumor vascular structure and permeability, thus reducing brain edema. Anti-angiogenic agents, therefore, may be useful alternatives to glucocorticoids in treating intractable brain edema. In recent years, several studies have described the efficacy of anti-angiogenic agents, especially bevacizumab. The first retrospective analysis of the use of bevacizumab in the treatment of radiation-induced brain necrosis and brain edema was published in 2007 (21). The results showed a significant improvement in radiation-induced brain edema in eight patients after four cycles of bevacizumab treatment. Furtner et al. (22). identified a total of 34 patients with recurrent WHO II and III meningiomas who had been treated at six European institutions. Compared to other types of therapy, such as cytotoxic chemotherapy, somatostatin analogs, and tyrosine kinase inhibitors, bevacizumab had the most pronounced inhibitory effect on anti-edematous activity. The volume of peritumoral brain edema in the bevacizumab-treated group was found to decrease by an average of 20.007 cm^3^ compared to the other treatment groups which increased by 0.107 cm^3^ on average. Some serious adverse reactions of bevacizumab should not be ignored, including gastrointestinal perforations, surgery and wound healing complications, hemorrhage, thromboembolism, proteinuria, and hypertension (23, 24). The efficacy of small molecular tyrosine kinase inhibitors has also been reported. Song et al. (25) documented a heavily treated breast cancer patient with intractable vasogenic brain edema after radiotherapy of BM. High dose steroids and dehydration produced no improvement. Surprisingly, the brain MRI demonstrated remarkable shrinkage of edema after taking apatinib for about one month. Another small molecular tyrosine kinase inhibitor, cediranib, has also been shown to reduce edema in a mouse glioblastoma model (26).

In hypoxia, tumor tissues produce VEGF, PDGF (Platelet derived growth factor) and FGF (Fibroblast growth factor), which can activate angiogenesis promoting signaling pathways (27). However, when VEGF-related signaling pathways are blocked, tumor tissues can upregulate the expression of other cytokines such as FGF and PDGF, through bypass activation and other mechanisms to maintain the tumor nutritional supply. Compared to other anti-angiogenic agents, anlotinib, which targets VEGFR, PDGFR, FGFR, and c-kit can inhibit three angiogenesis promoting signaling pathways (12). In addition, anlotinib can cross the blood-brain barrier and has a significantly lower incidence of grade 3 or other side-effects. It has been approved by the China NMPA (National Medical Products Administration) as a third-line therapy for advanced NSCLC. Phase III ALTER 0303 and Phase II ALTER 0302 trial evaluated the efficacy and safety of anlotinib in patients with advanced NSCLC (28, 29). A meta-analysis included 594 patients from three clinical studies (30). The incidence of adverse events except fatigue was higher in the anlotinib group than in the placebo group, but heterogeneity was insignificant among the other subgroups (hypertension, thyroid-stimulating hormone elevation, anorexia, hypertriglyceridemia, diarrhea, and hemoptysis). Adverse events identified to be associated with anlotinib were tolerable.

## Conclusion

This paper reported for the first time that anlotinib had achieved significant curative effect in the treatment of brain edema. From the two documents, we found that anlotinib is effective in the treatment of peritumoral brain edema. However, our understanding of anlotinib in the treatment of brain edema is just the tip of the iceberg, and the effect and mechanism remain to be verified before it can be extended to clinical practice.

## Data Availability Statement

The original contributions presented in the study are included in the article/supplementary material. Further inquiries can be directed to the corresponding authors.

## Ethics Statement

The studies involving human participants were reviewed and approved by the ethics committee of the Affiliated Hospital of Xuzhou Medical University. The patients/participants provided their written informed consent to participate in this study.

## Author Contributions

AJ, GL, and SY contributed to the design, analysis of this study, and writing of the manuscript. JS, MX, YW, AJ, GL, and SY were involved directly or indirectly in the care of patients. JS, MX, and YW were involved in the sample procurement. All authors contributed to the article and approved the submitted version.

## Funding

This study was partially supported by the XuZhou Clinical Technology Key Research Program (grant numbers 2018GG031).

## Conflict of Interest

The authors declare that the research was conducted in the absence of any commercial or financial relationships that could be construed as a potential conflict of interest.
